# Supplementary material for: Trans-differentiation of trophoblast stem cells: implications in placental biology
Source: Life Sci Alliance. 2022 Dec 27;6(3):e202201583. doi: 10.26508/lsa.202201583 (PMC9797987; doi:10.26508/lsa.202201583)
Supplement: Supplementary file 1 [file LSA-2022-01583_SdataF1.pdf]

D.

Percentage of CDH5-Ck positive cells

	TS	DIFF
Replicate1	0.2	11.6
Replicate2	3	9
Replicate3	1	14

E.

Percentage of PECAM1-Ck positive cells

Replicate1	0.1	36.2
Replicate2	0.2	44.7
Replicate3	0.5	28

F.

Percentage of ENG-Ck positive cells

Replicate1	0.2	35.4
Replicate2	0.1	30.1
Replicate3	0.5	20
