# Supplementary material for: Trans-differentiation of trophoblast stem cells: implications in placental biology
Source: Life Sci Alliance. 2022 Dec 27;6(3):e202201583. doi: 10.26508/lsa.202201583 (PMC9797987; doi:10.26508/lsa.202201583)
Supplement: Supplementary file 2 [file LSA-2022-01583_SdataFS1.pdf]

A.

	Percentage cell populationCD144-HLAG positive	
	Control	Induced
Replicate 1	34.5	57.2
Replicate 2	21	40.05
Replicate 3	25	42

B.

	Percentage cell population CD105-HLAG positive	
	Control	Induced
Replicate 1	17.8	30.06
Replicate 2	8.4	38
Replicate 3	20	35
