# Supplementary material for: Trans-differentiation of trophoblast stem cells: implications in placental biology
Source: Life Sci Alliance. 2022 Dec 27;6(3):e202201583. doi: 10.26508/lsa.202201583 (PMC9797987; doi:10.26508/lsa.202201583)
Supplement: Supplementary file 3 [file LSA-2022-01583_SdataFS2.pdf]

**A.**

| TS | Percentage cell population positive for Cdh5-Ck | |
|---|---|---|
| | $-VEGF_{165}$, bFGF | $+VEGF_{165}$, bFGF |
| Replicate 1 | 0.1 | 0.1 |
| Replicate 2 | 0.4 | 0.3 |
| Replicate 3 | 1 | 1.4 |

**C.**

| Diff | Percentage cell population positive for Cdh5-Ck | |
|---|---|---|
| | $-VEGF_{165}$, bFGF | $+VEGF_{165}$, bFGF |
| Replicate 1 | 14.3 | 38 |
| Replicate 2 | 11 | 35 |
| Replicate 3 | 15 | 37 |

**E.**

| Diff | Percentage cell population positive for Eng-Ck | |
|---|---|---|
| | $-VEGF_{165}$, bFGF | $+VEGF_{165}$, bFGF |
| Replicate 1 | 19.6 | 73.1 |
| Replicate 2 | 22 | 70 |
| Replicate 3 | 18 | 71 |
