# Supplementary material for: Trans-differentiation of trophoblast stem cells: implications in placental biology
Source: Life Sci Alliance. 2022 Dec 27;6(3):e202201583. doi: 10.26508/lsa.202201583 (PMC9797987; doi:10.26508/lsa.202201583)
Supplement: Supplementary file 4 [file LSA-2022-01583_TableS1.docx]

**Table S1: Differentiation induced differential expression in gene patterns related to endothelial cell function in trophoblast cells.**

| **Serial**  **No.** | **Gene**  **symbol** | **Accession No.** | **Control *C_t_* average** | **NOSTRIN *C_t_* average** | **Fold change** | **Gene description** |
| --- | --- | --- | --- | --- | --- | --- |
| 1 | Tnfsf10 | NM_009425 | 30.76 | 23.84 | 261.99 | Tumor necrosis factor(ligand) superfamily, member10 |
| 2 | Plat | NM_008872 | 27.87 | 22 | 126.74 | Plasminogen activator, tissue |
| 3 | Mmp1a | NM_032006 | 29.37 | 24.71 | 54.84 | Matrix metallopeptidase 1a (interstitial collagenase) |
| 4 | Pgf | NM_008827 | 30.39 | 26.31 | 36.67 | Placental growth factor |
| 5 | Cdh5 | NM_009868 | 24.29 | 21.37 | 16.38 | Cadherin 5 |
| 6 | Tfpi | NM_011576 | 19.63 | 17.75 | 7.96 | Tissue factor pathway inhibitor |
| 7 | Pecam1 | NM_008816 | 24.87 | 22.49 | 7.89 | Platelet/endothelial cell adhesion molecule 1 |
| 8 | Mmp2 | NM_008610 | 30.35 | 29.01 | 5.47 | Matrix metallopeptidase 2 |
| 9 | Sele | NM_011345 | 30.47 | 29.56 | 4.08 | Selectin, endothelial cell |
| 10 | Thbd | NM_009378 | 26.5 | 25.74 | 3.66 | Thrombomodulin |
| 11 | Kit | NM_021099 | 24.72 | 23.98 | 3.63 | Kit oncogene |
| 12 | Cx3cl1 | NM_009142 | 25.02 | 23.88 | 2.75 | Chemokine (C-X3-C motif) ligand 1 |
| 13 | Itgβ3 | NM_016780 | 22.25 | 21.92 | 2.72 | Integrin beta 3 |
| 14 | Fgf2 | NM_008006 | 22.2 | 32.15 | -455.9 | Fibroblast growth factor 2 |
| 15 | Il11 | NM_008350 | 24.25 | 31.22 | -57.73 | Interleukin 11 |
| 16 | Col18a1 | NM_009929 | 22.75 | 29.44 | -47.58 | Collagen, type XVIII, alpha 1 |
| 17 | Mmp9 | NM_013599 | 16.54 | 23.2 | -46.53 | Matrix metallopeptidase 9 |
| 18 | Kdr | NM_010612 | 19.82 | 26.17 | -37.65 | Kinase insert domain protein receptor |
| 19 | Ocln | NM_008756 | 23.47 | 29.42 | -28.53 | Occludin |
| 20 | Nos3 | NM_008713 | 28.85 | 34.78 | -28.04 | Nitric oxide synthase 3, endothelial cell |
| 21 | Edn1 | NM_010104 | 24.49 | 32.91 | -19.82 | Endothelin 1 |
| 22 | Flt1 | NM_010228 | 22.77 | 28.1 | -18.59 | FMS-like tyrosine kinase 1 |
| 23 | Bcl2 | NM_009741 | 21.62 | 26.42 | -12.86 | B-cell leukemia/lymphoma 2 |
| 24 | F3 | NM_010171 | 17.3 | 21.98 | -11.86 | Coagulation factor III |
| 25 | Tymp | NM_138302 | 27.77 | 32.27 | -10.4 | Thymidine phosphorylase |
| 26 | Cradd | NM_009950 | 22.4 | 26.78 | -9.6 | CASP2 and RIPK1 domain containing adaptor with death domain |
| 27 | Ptgis | NM_008968 | 18.26 | 22.62 | -9.41 | Prostaglandin I2 (prostacyclin) synthase |
| 28 | Ace | NM_009598 | 25.3 | 29.57 | -8.91 | Angiotensin I converting enzyme (peptidyl-dipeptidase A) 1 |
| 29 | Plau | NM_008873 | 25.78 | 29.98 | -8.51 | Plasminogen activator, urokinase |
| 30 | Cflar | NM_009805 | 20.1 | 24.28 | -8.35 | CASP8 and FADD-like apoptosis regulator |
| 31 | Agtr1a | NM_177322 | 29.98 | 34.1 | -8.03 | Angiotensin II receptor, type 1a |
| 32 | Vegfα | NM_009505 | 19.21 | 23.06 | -6.67 | Vascular endothelial growth factor A |
| 33 | Sod1 | NM_011434 | 14.68 | 18.5 | -6.53 | Superoxide dismutase 1, soluble |
| 34 | Il7 | NM_008371 | 28.13 | 31.87 | -6.17 | Interleukin 7 |
| 35 | Casp3 | NM_009810 | 22.08 | 25.75 | -5.86 | Caspase3 |
| 36 | Apoe | NM_009696 | 22.02 | 25.66 | -5.75 | Apolipoprotein E |
| 37 | Anxa5 | NM_009673 | 14.26 | 17.9 | -5.75 | Annexin A5 |
| 38 | Il1b | NM_008361 | 29.35 | 32.86 | -5.25 | Interleukin 1 beta |
| 39 | ItgaV | NM_008402 | 17.11 | 20.38 | -4.43 | Integrin alpha V |
| 40 | Itgβ1 | NM_010578 | 14.89 | 18.14 | -4.39 | Integrin beta 1 (fibronectin receptor beta) |
| 41 | Bcl2l1 | NM_009743 | 19.85 | 23.01 | -4.14 | Bcl2-like 1 |
| 42 | Tek | NM_013690 | 28.88 | 31.98 | -3.94 | Endothelial-specific receptor tyrosine kinase |
| 43 | Npr1 | NM_008727 | 24.27 | 27.34 | -3.85 | Natriuretic peptide receptor 1` |
| 44 | Cxcr5 | NM_007551 | 29.79 | 32.83 | -3.79 | Chemokine (C-X-C motif) receptor 5 |
| 45 | Bax | NM_007527 | 16.83 | 19.87 | -3.79 | Bcl2-associated X protein |
| 46 | F2rl1 | NM_007974 | 20.37 | 23.21 | -3.31 | Coagulation factor II (thrombin) receptor-like 1 |
| 47 | Serpine1 | NM_008871 | 17.89 | 20.63 | -3.08 | Serine (or cysteine) peptidase inhibitor, clade E, member 1 |
| 48 | Nppb | NM_008726 | 16.89 | 19.62 | -3.06 | Natriuretic peptide type B |
| 49 | Procr | NM_011171 | 16.19 | 18.88 | -2.97 | Protein C receptor, endothelial |
| 50 | Adam17 | NM_009615 | 19.47 | 22.06 | -2.77 | A disintegrin and metallopeptidase domain 17 |
| 51 | Hif1α | NM_001313919 | 17.41 | 19.89 | -2.58 | Hypoxia inducible factor 1, alpha subunit |
| 52 | Cxcl2 | NM_009140 | 32.57 | 26.79 | -2.58 | Chemokine (C-X-C motif) ligand 2 |
| 53 | Thbs1 | NM_011580 | 24.7 | 26.88 | -2.09 | Thrombospondin1 |
