# Supplementary material for: Trans-differentiation of trophoblast stem cells: implications in placental biology
Source: Life Sci Alliance. 2022 Dec 27;6(3):e202201583. doi: 10.26508/lsa.202201583 (PMC9797987; doi:10.26508/lsa.202201583)
Supplement: Supplementary file 5 [file LSA-2022-01583_SdataF3.pdf]

A.

Replicate1.	Cx3cl1 Ct	Cx3cl1 Ct mean	RPL7 Ct	RPL7 Ct mean	ΔCt	ΔΔCt	RQ(2^-ΔΔCt)
TS	24.94	24.94333333	15.58	15.57666667	9.366667	0	**1**
	24.94		15.57				
	24.95		15.58				
Diff	23.81	23.81333333	15.96	15.94	7.873333	-1.49333	**2.815387168**
	23.82		15.9				
	23.81		15.96				

Replicate2.	Cx3cl1 Ct	Cx3cl1 Ct mean	RPL7 Ct	RPL7 Ct mean	ΔCt	ΔΔCt	RQ(2^-ΔΔCt)
TS	24.98	24.97	15.65	15.63	9.34	0	**1**
	24.97		15.6				
	24.96		15.64				
Diff	23.88	23.87666667	15.91	15.91	7.966667	-1.37333	**2.590684504**
	23.87		15.9				
	23.88		15.92				

Replicate3.	Cx3cl1 Ct	Cx3cl1 Ct mean	RPL7 Ct	RPL7 Ct mean	ΔCt	ΔΔCt	RQ(2^-ΔΔCt)
TS	25	25.02	15.56	15.55333333	9.466667	0	**1**
	25.02		15.5				
	25.04		15.6				
Diff	23.95	23.94	15.95	15.93	8.01	-1.45667	**2.744734621**
	23.95		15.9				
	23.92		15.94				

Replicate1.	C-kit Ct	C-kit Ct mean	RPL7 Ct	RPL7 Ct mean	ΔCt	ΔΔCt	RQ(2^-ΔΔCt)
TS	26.6	26.56666667	15.55	15.55666667	11.01	0	**1**
	26.5		15.57				
	26.6		15.55				
Diff	21.9	21.91	15.63	15.62333333	6.286667	-4.72333	**26.41587573**
	21.92		15.6				
	21.91		15.64				

Replicate2.	C-kit Ct	C-kit Ct mean	RPL7 Ct	RPL7 Ct mean	ΔCt	ΔΔCt	RQ(2^-ΔΔCt)
TS	26.3	26.2	15.45	15.43333333	10.76667	0	**1**
	26.3		15.45				
	26		15.4				
Diff	21.89	21.88666667	15.86	15.84666667	6.04	-4.72667	**26.47697993**
	21.88		15.8				
	21.89		15.88				

A. Cont.

**Replicate3.**	**C-kit Ct**	**C-kit Ct mean**	**RPL7 Ct**	**RPL7 Ct mean**	**ΔCt**	**ΔΔCt**	**RQ(2^-ΔΔCt)**
TS	26.5	26.52	15.91	15.9	10.62	0	**1**
	26.52		15.9				
	26.54		15.89				
Diff	21.5	21.54	15.5	15.53666667	6.003333	-4.61667	**24.53325351**
	21.52		15.6				
	21.6		15.51				

**Replicate1.**	**Mmp9 Ct**	**Mmp9 Ct mean**	**RPL7 Ct**	**RPL7 Ct mean**	**ΔCt**	**ΔΔCt**	**RQ(2^-ΔΔCt)**
TS	22.05	22.05	16	16.01666667	6.033333	0	**1**
	22		16.05				
	22.1		16				
Diff	25.76	25.76	16.46	16.44	9.32	3.286667	**0.10247425**
	25.77		16.42				
	25.75		16.44				

**Replicate2.**	**Mmp9 Ct**	**Mmp9 Ct mean**	**RPL7 Ct**	**RPL7 Ct mean**	**ΔCt**	**ΔΔCt**	**RQ(2^-ΔΔCt)**
TS	22.06	22.06666667	16.01	16.02666667	6.04	0	**1**
	22.08		16.05				
	22.06		16.02				
Diff	25.74	25.74666667	16.7	16.73333333	9.013333	2.973333	**0.127331976**
	25.74		16.75				
	25.76		16.75				
**Replicate3.**	**Mmp9 Ct**	**Mmp9 Ct mean**	**RPL7 Ct**	**RPL7 Ct mean**	**ΔCt**	**ΔΔCt**	**RQ(2^-ΔΔCt)**
TS	22.21	22.22333333	16.04	16.03333333	6.19	0	**1**
	22.24		16				
	22.22		16.06				
Diff	25.77	25.77666667	16.92	16.94	8.836667	2.646667	**0.159688612**
	25.77		16.94				
	25.79		16.96				

**Replicate1.**	**Plau Ct**	**Plau Ct mean**	**RPL7 Ct**	**RPL7 Ct mean**	**ΔCt**	**ΔΔCt**	**RQ(2^-ΔΔCt)**
TS	28.91	28.91	15.27	15.27	13.64	0	**1**
	28.92		15.28				
	28.9		15.26				
Diff	29.71	29.72	15.51	15.51666667	14.20333	0.563333	**0.676736762**
	29.7		15.54				
	29.75		15.5				

Cont.

A.

Replicate2.	Plau Ct	Plau Ct mean	RPL7 Ct	RPL7 Ct mean	ΔCt	ΔΔCt	RQ(2^-ΔΔCt)
TS	28.84	28.84	15.29	15.29	13.55	0	**1**
	28.86		15.3				
	28.82		15.28				
Diff	29.77	29.77666667	15.44	15.43333333	14.34333	0.793333	**0.577009376**
	29.79		15.4				
	29.77		15.46				

Replicate3.	Plau Ct	Plau Ct mean	RPL7 Ct	RPL7 Ct mean	ΔCt	ΔΔCt	RQ(2^-ΔΔCt)
TS	28.76	28.75333333	15.33	15.32333333	13.43	0	**1**
	28.74		15.3				
	28.76		15.34				
Diff	29.44	29.44666667	15.86	15.86	13.58667	0.156667	**0.897095409**
	29.48		15.88				
	29.42		15.84				

Replicate1.	Kdr Ct	Kdr Ct mean	RPL7 Ct	RPL7 Ct mean	ΔCt	ΔΔCt	RQ(2^-ΔΔCt)
TS	23.22	23.22	15.34	15.32	7.9	0	**1**
	23.2		15.3				
	23.24		15.32				
Diff	26.33	26.32	15.96	15.96	10.36	2.46	**0.181746565**
	26.3		15.98				
	26.33		15.94				

Replicate2.	Kdr Ct	Kdr Ct mean	RPL7 Ct	RPL7 Ct mean	ΔCt	ΔΔCt	RQ(2^-ΔΔCt)
TS	23.21	23.21	15.4	15.44666667	7.763333	0	**1**
	23.22		15.46				
	23.2		15.48				
Diff	26.38	26.35333333	15.98	15.98333333	10.37	2.606667	**0.164178069**
	26.36		15.99				
	26.32		15.98				

Replicate3.	Kdr Ct	Kdr Ct mean	RPL7 Ct	RPL7 Ct mean	ΔCt	ΔΔCt	RQ(2^-ΔΔCt)
TS	23.41	23.44666667	15.3	15.32	8.126667	0	**1**
	23.45		15.34				
	23.48		15.32				
Diff	26.4	26.44666667	15.97	15.97	10.47667	2.35	**0.196146024**
	26.48		15.98				
	26.46		15.96				

B.

Replicate1.	Cdh5 Ct	Cdh5 Ct mean	RPL7 Ct	RPL7 Ct mean	ΔCt	ΔΔCt	RQ(2^-ΔΔCt)
TS	27.92	27.94666667	15.93	15.94333333	12.00333	0	**1**
	27.94		15.94				
	27.98		15.96				
Diff	20.79	20.78333333	16.4	16.41	4.373333	-7.63	**198.0883192**
	20.77		16.43				
	20.79		16.4				
**Replicate2.**	**Cdh5 Ct**	**Cdh5 Ct mean**	**RPL7 Ct**	**RPL7 Ct mean**	**ΔCt**	**ΔΔCt**	**RQ(2^-ΔΔCt)**
TS	27.76	27.75333333	15.91	15.92	11.83333	0	**1**
	27.72		15.95				
	27.78		15.9				
Diff	20.77	20.77333333	16.48	16.47333333	4.3	-7.53333	**185.2504624**
	20.78		16.46				
	20.77		16.48				
**Replicate3.**	**Cdh5 Ct**	**Cdh5 Ct mean**	**RPL7 Ct**	**RPL7 Ct mean**	**ΔCt**	**ΔΔCt**	**RQ(2^-ΔΔCt)**
TS	28.27	28.27666667	15.95	15.93	12.34667	0	**1**
	28.3		15.94				
	28.26		15.9				
Diff	20.75	20.74333333	16	16.08333333	4.66	-7.68667	**206.0237241**
	20.76		16.1				
	20.72		16.15				
**Replicate1.**	**Pecam1 Ct**	**Pecam1 Ct mean**	**RPL7 Ct**	**RPL7 Ct mean**	**ΔCt**	**ΔΔCt**	**RQ(2^-ΔΔCt)**
TS	24.72	24.74	15.43	15.42666667	9.313333	0	**1**
	24.74		15.42				
	24.76		15.43				
Diff	22.44	22.42	15.95	15.94333333	6.476667	-2.83667	**7.143676088**
	22.4		15.95				
	22.42		15.93				
**Replicate2.**	**Pecam1 Ct**	**Pecam1 Ct mean**	**RPL7 Ct**	**RPL7 Ct mean**	**ΔCt**	**ΔΔCt**	**RQ(2^-ΔΔCt)**
TS	24.91	24.91	15.42	15.41333333	9.496667	0	**1**
	24.92		15.42				
	24.9		15.4				
Diff	22.53	22.53	15.9	15.92666667	6.603333	-2.89333	**7.42985128**
	22.54		15.94				
	22.52		15.94				

Cont

B.

Replicate3.	Pecam1 Ct	Pecam1 Ct mean	RPL7 Ct	RPL7 Ct mean	ΔCt	ΔΔCt	RQ(2^-ΔΔCt)
TS	24.97	24.97333333	15.24	15.21333333	9.76	0	**1**
	24.98		15.2				
	24.97		15.2				
Diff	22.51	22.51	15.96	15.95333333	6.556667	-3.20333	**9.210843842**
	22.5		15.94				
	22.52		15.96				

Replicate1.	Itgβ3 Ct	Itgβ3 Ct mean	RPL7 Ct	RPL7 Ct mean	ΔCt	ΔΔCt	RQ(2^-ΔΔCt)
TS	28.58	28.57333333	15	15.08666667	13.48667	0	**1**
	28.56		15.12				
	28.58		15.14				
Diff	21.72	21.74	15.9	15.92	5.82	-7.66667	**203.1873347**
	21.74		15.92				
	21.76		15.94				

Replicate2.	Itgβ3 Ct	Itgβ3 Ct mean	RPL7 Ct	RPL7 Ct mean	ΔCt	ΔΔCt	RQ(2^-ΔΔCt)
TS	28.64	28.64	15.88	15.88	12.76	0	**1**
	28.66		15.86				
	28.62		15.9				
Diff	21.4	21.42	16.4	16.42	5	-7.76	**216.7668**
	21.42		16.42				
	21.44		16.44				

Replicate3.	Itgβ3 Ct	Itgβ3 Ct mean	RPL7 Ct	RPL7 Ct mean	ΔCt	ΔΔCt	RQ(2^-ΔΔCt)
TS	28.4	28.40666667	15.2	15.16666667	13.24	0	**1**
	28.42		15.2				
	28.4		15.1				
Diff	21.24	21.25666667	15.96	15.95333333	5.303333	-7.93667	**245.0048786**
	21.3		15.94				
	21.23		15.96				

Replicate1.	Col18a1 Ct	Col18a1 Ct mean	RPL7 Ct	RPL7 Ct mean	ΔCt	ΔΔCt	RQ(2^-ΔΔCt)
TS	24.99	24.98666667	15	15.14666667	9.84	0	**1**
	24.98		15.2				
	24.99		15.24				
Diff	28.96	27.60666667	15.4	15.41	12.19667	2.356667	**0.195241728**
	28.94		15.42				
	24.92		15.41				

Cont

B.

Replicate2.	Col18a1 Ct	Col18a1 Ct mean	RPL7 Ct	RPL7 Ct mean	ΔCt	ΔΔCt	RQ(2^-ΔΔCt)
TS	24.96	24.94	15.3	15.30666667	9.633333	0	**1**
	24.94		15.32				
	24.92		15.3				
Diff	28.97	28.97	15.9	15.91	13.06	3.426667	**0.092997345**
	28.98		15.92				
	28.96		15.91				

Replicate3.	Col18a1 Ct	Col18a1 Ct mean	RPL7 Ct	RPL7 Ct mean	ΔCt	ΔΔCt	RQ(2^-ΔΔCt)
TS	25	25.1	15.4	15.40666667	9.693333	0	**1**
	25.2		15.42				
	25.1		15.4				
Diff	29	29.11666667	15.8	15.81	13.30667	3.613333	**0.081710578**
	29.2		15.82				
	29.15		15.81				

C.

Replicate1.	Tnsf10 Ct	Tnsf10 Ct mean	RPL7 Ct	RPL7 Ct mean	ΔCt	ΔΔCt	RQ(2^-ΔΔCt)
TS	31	31.14666667	15.21	15.212	15.93467	0	**1**
	31.2		15.212				
	31.24		15.214				
Diff	17	17.06666667	15.28	15.27666667	1.79	-14.1447	**18112.10576**
	17.2		15.27				
	17		15.28				

Replicate2.	Tnsf10 Ct	Tnsf10 Ct mean	RPL7 Ct	RPL7 Ct mean	ΔCt	ΔΔCt	RQ(2^-ΔΔCt)
TS	32	32.03333333	15	15.07333333	16.96	0	**1**
	32		15.1				
	32.1		15.12				
Diff	18	18.07333333	15.9	15.91	2.163333	-14.7967	**28460.36742**
	18.1		15.92				
	18.12		15.91				

Replicate3.	Tnsf10 Ct	Tnsf10 Ct mean	RPL7 Ct	RPL7 Ct mean	ΔCt	ΔΔCt	RQ(2^-ΔΔCt)
TS	31.7	31.70666667	15.3	15.32666667	16.38	0	**1**
	31.72		15.36				
	31.7		15.32				
Diff	17.64	17.62666667	15.4	15.40333333	2.223333	-14.1567	**18263.3863**
	17.6		15.41				
	17.64		15.4				

C.

**Replicate1.**	**Bcl2 Ct**	**Bcl2 Ct mean**	**RPL7 Ct**	**RPL7 Ct mean**	**ΔCt**	**ΔΔCt**	**RQ(2^-ΔΔCt)**
TS	28.15	28.15333333	14.57	14.57333333	13.58	0	**1**
	28.16		14.57				
	28.15		14.58				
Diff	29.83	29.82666667	14.87	14.87	14.95667	1.376667	**0.385107556**
	29.82		14.88				
	29.83		14.86				

**Replicate2.**	**Bcl2 Ct**	**Bcl2 Ct mean**	**RPL7 Ct**	**RPL7 Ct mean**	**ΔCt**	**ΔΔCt**	**RQ(2^-ΔΔCt)**
TS	28.37	28.37	14.65	14.65333333	13.71667	0	**1**
	28.38		14.66				
	28.36		14.65				
Diff	29.92	29.91666667	14.86	14.86666667	15.05	1.333333	**0.396850263**
	29.91		14.86				
	29.92		14.88				

**Replicate3.**	**Bcl2 Ct**	**Bcl2 Ct mean**	**RPL7 Ct**	**RPL7 Ct mean**	**ΔCt**	**ΔΔCt**	**RQ(2^-ΔΔCt)**
TS	28.36	28.35333333	14.6	14.61	13.74333	0	**1**
	28.34		14.62				
	28.36		14.61				
Diff	29.91	29.90666667	14.85	14.84333333	15.06333	1.32	**0.400534939**
	29.9		14.83				
	29.91		14.85				

**Replicate1.**	**Cradd Ct**	**Cradd Ct mean**	**RPL7 Ct**	**RPL7 Ct mean**	**ΔCt**	**ΔΔCt**	**RQ(2^-ΔΔCt)**
TS	26.9	26.86333333	15.43	15.43333333	11.43	0	**1**
	26.8		15.44				
	26.89		15.43				
Diff	28.28	28.27333333	15.99	15.98666667	12.28667	0.856667	**0.552227**
	28.26		15.98				
	28.28		15.99				

**Replicate2.**	**Cradd Ct**	**Cradd Ct mean**	**RPL7 Ct**	**RPL7 Ct mean**	**ΔCt**	**ΔΔCt**	**RQ(2^-ΔΔCt)**
TS	27	27.07333333	15.48	15.14	11.93333	0	**1**
	27.1		15.46				
	27.12		14.48				
Diff	28.29	28.29	15.94	15.94333333	12.34667	0.413333	**0.750886452**
	28.28		15.95				
	28.3		15.94				

Cont

C.

**Replicate3.**	**Cradd Ct**	**Cradd Ct mean**	**RPL7 Ct**	**RPL7 Ct mean**	**ΔCt**	**ΔΔCt**	**RQ(2^-ΔΔCt)**
TS	26.95	26.95	15.47	15.47333333	11.47667	0	**1**
	26.94		15.46				
	26.96		15.49				
Diff	28.36	28.36333333	15.5	15.50333333	12.86	1.383333	**0.383332086**
	28.37		15.51				
	28.36		15.5				

**Replicate1.**	**Casp3 Ct**	**Casp3 Ct mean**	**RPL7 Ct**	**RPL7 Ct mean**	**ΔCt**	**ΔΔCt**	**RQ(2^-ΔΔCt)**
TS	24.5	24.51	15.16	15.16333333	9.346667	0	**1**
	24.52		15.17				
	24.51		15.16				
Diff	26	26.01666667	15.86	15.86333333	10.15333	0.806667	**0.571701243**
	26.03		15.87				
	26.02		15.86				
**Replicate2.**	**Casp3 Ct**	**Casp3 Ct mean**	**RPL7 Ct**	**RPL7 Ct mean**	**ΔCt**	**ΔΔCt**	**RQ(2^-ΔΔCt)**
TS	24.92	24.94	15.18	15.17333333	9.766667	0	**1**
	24.94		15.16				
	24.96		15.18				
Diff	26.04	26.05	15.79	15.78666667	10.26333	0.496667	**0.708742434**
	26.05		15.8				
	26.06		15.77				
**Replicate3.**	**Casp3 Ct**	**Casp3 Ct mean**	**RPL7 Ct**	**RPL7 Ct mean**	**ΔCt**	**ΔΔCt**	**RQ(2^-ΔΔCt)**
TS	24.23	24.23	15.32	15.33	8.9	0	**1**
	24.24		15.34				
	24.22		15.33				
Diff	25.8	25.66666667	15.9	15.91	9.756667	0.856667	**0.552227**
	25.6		15.91				
	25.6		15.92				
