# Supplementary material for: Trans-differentiation of trophoblast stem cells: implications in placental biology
Source: Life Sci Alliance. 2022 Dec 27;6(3):e202201583. doi: 10.26508/lsa.202201583 (PMC9797987; doi:10.26508/lsa.202201583)
Supplement: Supplementary file 6 [file LSA-2022-01583_SdataF4.pdf]

Figure 4A

Original blots

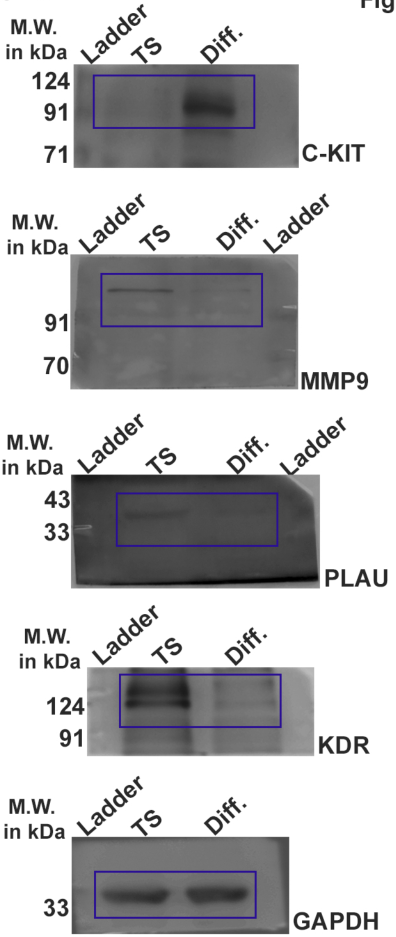

Figure 4A

Cropped blots

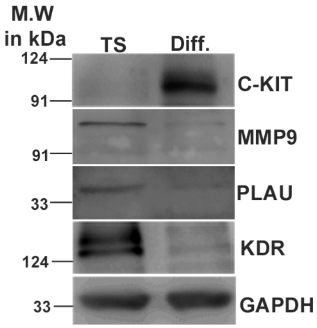

Original blots

Figure 4C.

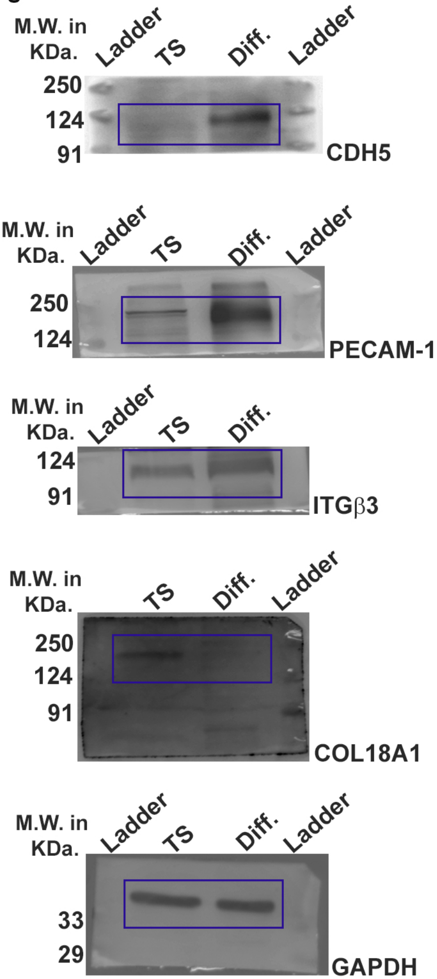

Cropped blots

Figure 4C.

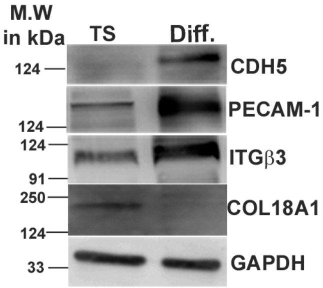

Original blots

Figure 4E.

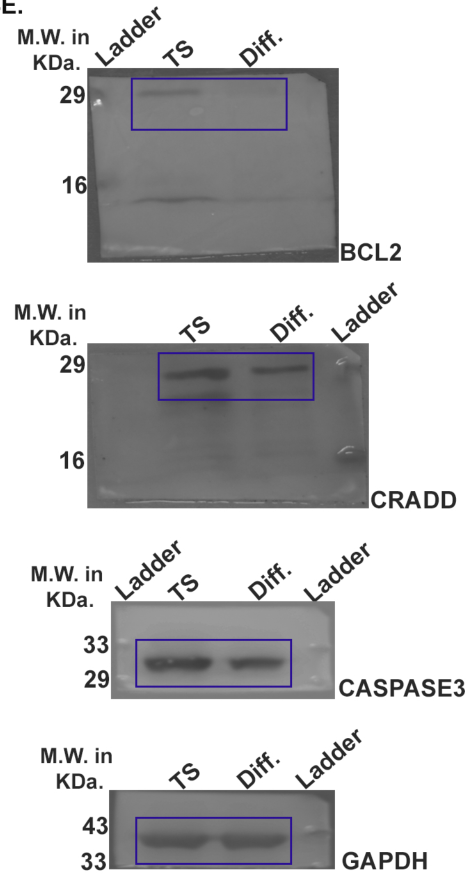

Cropped blots

Figure 4E.

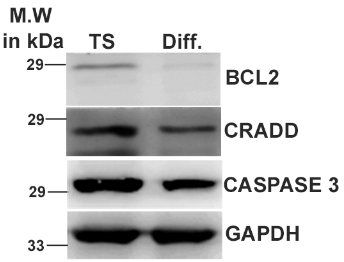

G.

	TNSF10/total protein(pg/ml)	
	TS	Diff
Replicate1	3.515113	41.08557
Replicate2	2.800619	42.06567
Replicate3	3.157866	48.92638
Avg	3.16	44.02

H.

	CX3CL1/total protein(ng/ml)	
	TS	Diff
Replicate1	15.08297	34.0647
Replicate2	13.22235	30.24152
Replicate3	15.24336	33.93286
Avg	14.52	32.74
