# Supplementary material for: Trans-differentiation of trophoblast stem cells: implications in placental biology
Source: Life Sci Alliance. 2022 Dec 27;6(3):e202201583. doi: 10.26508/lsa.202201583 (PMC9797987; doi:10.26508/lsa.202201583)

Original blots

Figure 5A.

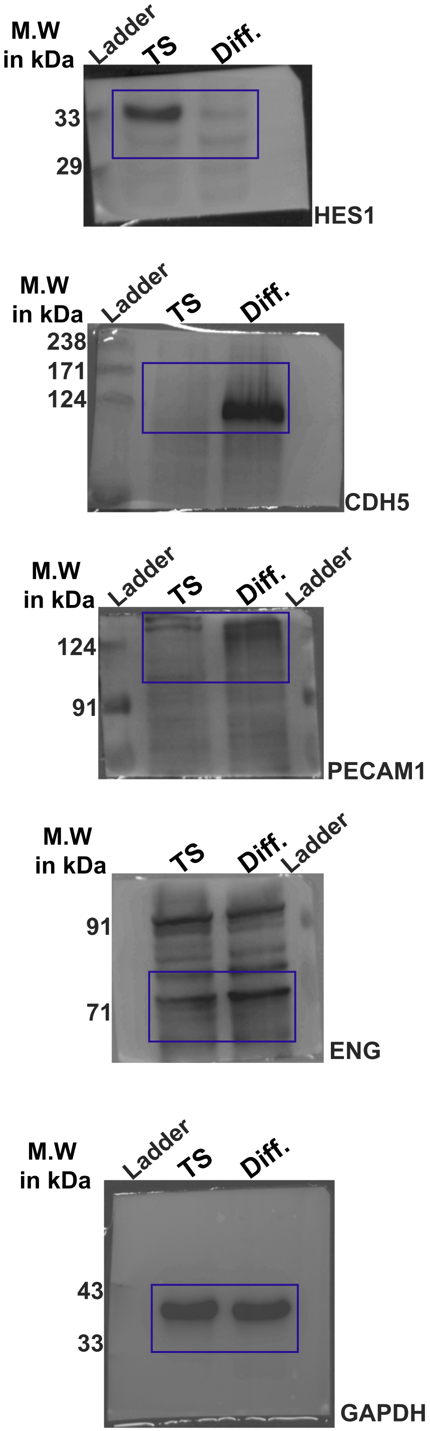

Cropped blots

Figure 5A.

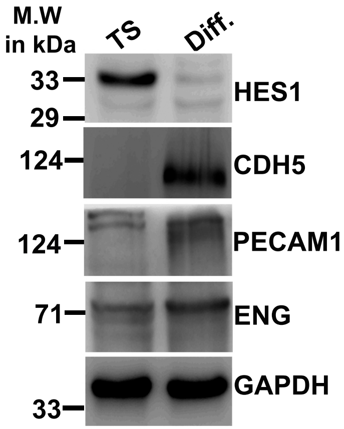

C.

**Replicate1.**	Hes1 Ct	Hes1 Ct mean	RPL7 Ct	RPL7 Ct mean	ΔCt	ΔΔCt	RQ(2^-ΔΔCt)
Scramble	25.26	25.26633333	15.4	15.40266667	9.863667	0	**1**
	25.269		15.405				
	25.27		15.403				
Hes1 siRNA	26.26	26.24066667	15.04	15.02866667	11.212	1.348333	**0.392745504**
	26.262		15				
	26.2		15.046				

**Replicate2.**	Hes1 Ct	Hes1 Ct mean	RPL7 Ct	RPL7 Ct mean	ΔCt	ΔΔCt	RQ(2^-ΔΔCt)
Scramble	25.61	25.61166667	15.37	15.376	10.23567	0	**1**
	25.612		15.378				
	25.613		15.38				
Hes1 siRNA	26.5	26.51666667	15.17	15.17333333	11.34333	1.107667	**0.464043943**
	26.52		15.18				
	26.53		15.17				

**Replicate3.**	Hes1 Ct	Hes1 Ct mean	RPL7 Ct	RPL7 Ct mean	ΔCt	ΔΔCt	RQ(2^-ΔΔCt)
Scramble	25.36	25.36166667	15.64	15.63366667	9.728	0	**1**
	25.362		15.63				
	25.363		15.631				
Hes1 siRNA	26.54	26.541	15.5	15.504	11.037	1.309	**0.403600537**
	26.543		15.505				
	26.54		15.507				

D.

**Replicate1.**	Cdh5 Ct	Cdh5 Ct mean	RPL7 Ct	RPL7 Ct mean	ΔCt	ΔΔCt	RQ(2^-ΔΔCt)
Scramble	23.83	23.83133333	15.966	15.96366667	7.867667	0	**1**
	23.834		15.96				
	23.83		15.965				
Hes1 siRNA	23.112	23.10733333	15.77	15.772	7.335333	-0.53	**1.44626641**
	23.11		15.776				
	23.1		15.77				

**Replicate2.**	Cdh5 Ct	Cdh5 Ct mean	RPL7 Ct	RPL7 Ct mean	ΔCt	ΔΔCt	RQ(2^-ΔΔCt)
Scramble	23.98	23.98033333	15.91	15.91133333	8.069	0	**1**
	23.981		15.914				
	23.98		15.91				
Hes1 siRNA	23.31	23.31433333	15.76	15.761	7.553333	-0.52	**1.42965463**
	23.313		15.763				
	23.32		15.76				

**Replicate3.**	Cdh5 Ct	Cdh5 Ct mean	RPL7 Ct	RPL7 Ct mean	ΔCt	ΔΔCt	RQ(2^-ΔΔCt)
Scramble	23.9	23.906	15.94	15.94033333	7.965667	0	1
	23.908		15.94				
	23.91		15.941				
Hes1 siRNA	23.21	23.214	15.76	15.76633333	7.447667	-0.52	**1.43196874**
	23.212		15.77				
	23.22		15.769				

E.

**Replicate1.**	Eng Ct	Eng Ct mean	RPL7 Ct	RPL7 Ct mean	ΔCt	ΔΔCt	RQ(2^-ΔΔCt)
Scramble	26.74	26.741	15.911	15.91366667	10.82733	0	**1**
	26.743		15.91				
	26.74		15.92				
Hes1 siRNA	25.94	25.9416667	15.81	15.814	10.12767	-0.6997	**1.6241295**
	25.945		15.812				
	25.94		15.82				

**Replicate2.**	Eng Ct	Eng Ct mean	RPL7 Ct	RPL7 Ct mean	ΔCt	ΔΔCt	RQ(2^-ΔΔCt)
Scramble	26.9	26.9056667	15.91	15.91566667	10.99	0	**1**
	26.907		15.917				
	26.91		15.92				
Hes1 siRNA	26.15	26.1523333	15.68	15.681	10.47133	-0.5187	**1.4326306**
	26.15		15.683				
	26.157		15.68				

**Replicate3.**	Eng Ct	Eng Ct mean	RPL7 Ct	RPL7 Ct mean	ΔCt	ΔΔCt	RQ(2^-ΔΔCt)
Scramble	26.89	26.8963333	15.9	15.90033333	10.996	0	**1**
	26.899		15.901				
	26.9		15.9				
Hes1 siRNA	26.54	26.5286667	15.94	15.94033333	10.58833	-0.4077	**1.32653861**
	26.546		15.941				
	26.5		15.94				

Original blots

Figure 5F.

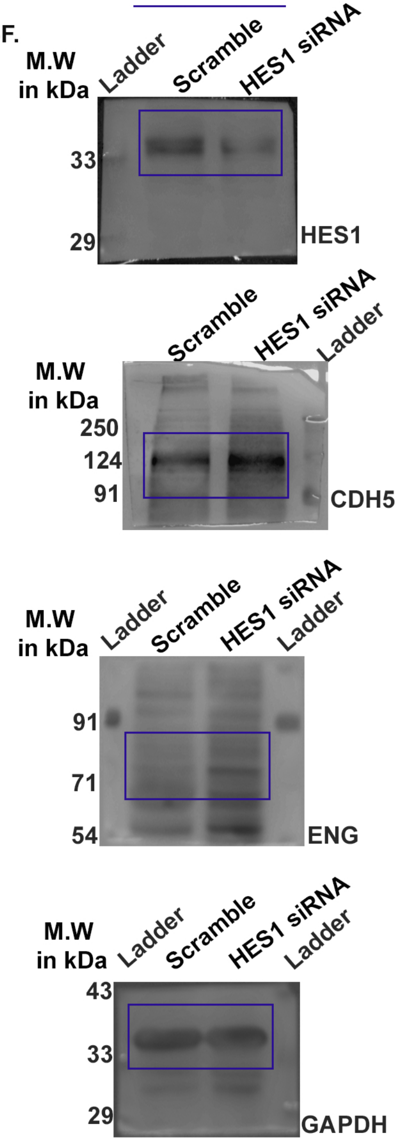

Cropped blots

Figure 5F.

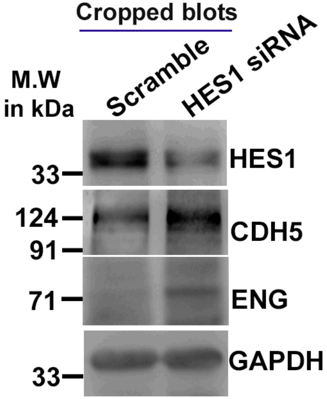

Supplement: Supplementary file 7 [file LSA-2022-01583_SdataF5.pdf]
