# Supplementary figures and images for: Trans-differentiation of trophoblast stem cells: implications in placental biology
Source: Life Sci Alliance. 2022 Dec 27;6(3):e202201583. doi: 10.26508/lsa.202201583 (PMC9797987; doi:10.26508/lsa.202201583)

### Original blots

### Cropped blots

Figure S3.

Figure S3.

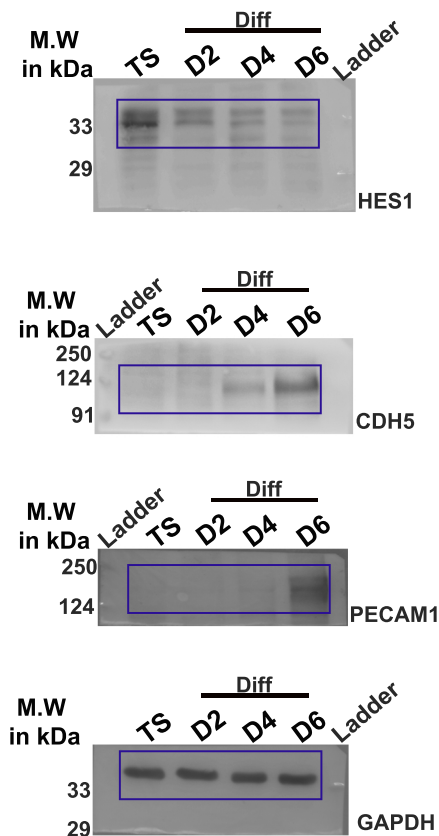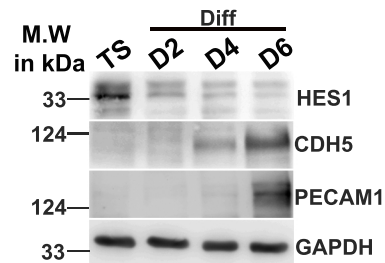

Supplement: Supplementary file 8 [file LSA-2022-01583_SdataFS3.pdf]
