# Supplementary material for: Trans-differentiation of trophoblast stem cells: implications in placental biology
Source: Life Sci Alliance. 2022 Dec 27;6(3):e202201583. doi: 10.26508/lsa.202201583 (PMC9797987; doi:10.26508/lsa.202201583)
Supplement: Supplementary file 9 [file LSA-2022-01583_SdataFS4.pdf]

A.

Percentage cell population CDH5 positive	TS	Diff
Replicate 1	0.1	10.7
Replicate 2	0.4	10.5
Replicate 3	0.5	10.8

Percentage cell population HES1 positive	TS	Diff
Replicate 1	26.2	1.4
Replicate 2	26.5	2
Replicate 3	26.8	2.2

Percentage cell population CDH5-HES1 positive	TS	Diff
Replicate 1	0.1	2.8
Replicate 2	0.2	2.5
Replicate 3	0.3	2.4
