# Supplementary material for: Trans-differentiation of trophoblast stem cells: implications in placental biology
Source: Life Sci Alliance. 2022 Dec 27;6(3):e202201583. doi: 10.26508/lsa.202201583 (PMC9797987; doi:10.26508/lsa.202201583)
Supplement: Supplementary file 10 [file LSA-2022-01583_SdataFS5.pdf]

20nM

Replicate1.	Hes1 Ct	Hes1 Ct mean	RPL7 Ct	RPL7 Ct mean	ΔCt	ΔΔCt	RQ(2^-ΔΔCt)
Scramble	25.25	25.256	15.398	15.396	9.86	0	**1**
	25.26		15.39				
	25.258		15.4				
Hes1 siRNA	25.89	25.896	15.74	15.74533333	10.15067	0.2907	**0.817524195**
	25.898		15.746				
	25.9		15.75				

Replicate2.	Hes1 Ct	Hes1 Ct mean	RPL7 Ct	RPL7 Ct mean	ΔCt	ΔΔCt	RQ(2^-ΔΔCt)
Scramble	25.6	25.60666667	15.36	15.36566667	10.241	0	**1**
	25.62		15.367				
	25.6		15.37				
Hes1 siRNA	25.92	25.92633333	15.93	15.93333333	9.993	-0.248	**1.187559666**
	25.93		15.934				
	25.929		15.936				

Replicate3.	Hes1 Ct	Hes1 Ct mean	RPL7 Ct	RPL7 Ct mean	ΔCt	ΔΔCt	RQ(2^-ΔΔCt)
Scramble	25.35	25.33466667	15.61	15.616	9.718667	0	**1**
	25.354		15.62				
	25.3		15.618				
Hes1 siRNA	25.85	25.85633333	15.71	15.71133333	10.145	0.4263	**0.744150672**
	25.86		15.714				
	25.859		15.71				

				100nM			
**Replicate1.**	Hes1 Ct	Hes1 Ct mean	RPL7 Ct	RPL7 Ct mean	ΔCt	ΔΔCt	RQ(2^-ΔΔCt)
Scramble	25.25	25.256	15.398	15.396	9.86	0	**1**
	25.26		15.39				
	25.258		15.4				
Hes1 siRNA	26.5	26.51666667	15.17	15.17333333	11.34333	1.107667	**0.464043943**
	26.52		15.18				
	26.53		15.17				
**Replicate2.**	Hes1 Ct	Hes1 Ct mean	RPL7 Ct	RPL7 Ct mean	ΔCt	ΔΔCt	RQ(2^-ΔΔCt)
Scramble	25.6	25.60666667	15.36	15.36566667	10.241	0	**1**
	25.62		15.367				
	25.6		15.37				
Hes1 siRNA	26.26	26.24066667	15.04	15.02866667	11.212	1.348333	**0.392745504**
	26.262		15				
	26.2		15.046				
**Replicate3.**	Hes1 Ct	Hes1 Ct mean	RPL7 Ct	RPL7 Ct mean	ΔCt	ΔΔCt	RQ(2^-ΔΔCt)
Scramble	25.35	25.33466667	15.61	15.616	9.718667	0	**1**
	25.354		15.62				
	25.3		15.618				
Hes1 siRNA	26.54	26.541	15.5	15.504	11.037	1.309	**0.403600537**
	26.543		15.505				
	26.54		15.507				

				200nM			
**Replicate1.**	Hes1 Ct	Hes1 Ct mean	RPL7 Ct	RPL7 Ct mean	ΔCt	ΔΔCt	RQ(2^-ΔΔCt)
Scramble	25.25	25.256	15.398	15.396	9.86	0	**1**
	25.26		15.39				
	25.258		15.4				
Hes1 siRNA	25.99	25.98933333	15.69	15.69366667	10.29567	0.435667	**0.73935202**
	25.98		15.7				
	25.998		15.691				
**Replicate2.**	Hes1 Ct	Hes1 Ct mean	RPL7 Ct	RPL7 Ct mean	ΔCt	ΔΔCt	RQ(2^-ΔΔCt)
Scramble	25.6	25.60666667	15.36	15.36566667	10.241	0	**1**
	25.62		15.367				
	25.6		15.37				
Hes1 siRNA	26.22	26.226	15.66	15.66066667	10.56533	0.324333	**0.79866736**
	26.228		15.662				
	26.23		15.66				
**Replicate3.**	Hes1 Ct	Hes1 Ct mean	RPL7 Ct	RPL7 Ct mean	ΔCt	ΔΔCt	RQ(2^-ΔΔCt)
Scramble	25.35	25.33466667	15.61	15.616	9.718667	0	**1**
	25.354		15.62				
	25.3		15.618				
Hes1 siRNA	25.93	25.93066667	15.34	15.343	10.58767	0.869	**0.54752624**
	25.932		15.343				
	25.93		15.346				
