# Supplementary material for: Trans-differentiation of trophoblast stem cells: implications in placental biology
Source: Life Sci Alliance. 2022 Dec 27;6(3):e202201583. doi: 10.26508/lsa.202201583 (PMC9797987; doi:10.26508/lsa.202201583)
Supplement: Supplementary file 11 [file LSA-2022-01583_SdataFS6.pdf]

A.

	Percentage cell population positive for CDH5-Ck	
**Scramble siRNA**	-$VEGF_{165}$, bFGF	+$VEGF_{165}$, bFGF
Replicate 1	1.4	1.2
Replicate 2	1.5	1.4
Replicate 3	1.8	1.5
**Hes1 siRNA**	-$VEGF_{165}$, bFGF	+$VEGF_{165}$, bFGF
Replicate 1	3.2	7
Replicate 2	4	7.5
Replicate 3	3.8	8

C.

	Percentage cell population positive for ENG-Ck	
**Scramble siRNA**	-$VEGF_{165}$, bFGF	+$VEGF_{165}$, bFGF
Replicate 1	1.1	0.5
Replicate 2	1.2	0.8
Replicate 3	1.5	1
**Hes1 siRNA**	-$VEGF_{165}$, bFGF	+$VEGF_{165}$, bFGF
Replicate 1	15.7	28.1
Replicate 2	16	29
Replicate 3	16.2	30
