# Supplementary material for: Trans-differentiation of trophoblast stem cells: implications in placental biology
Source: Life Sci Alliance. 2022 Dec 27;6(3):e202201583. doi: 10.26508/lsa.202201583 (PMC9797987; doi:10.26508/lsa.202201583)
Supplement: Supplementary file 12 [file LSA-2022-01583_SdataF6.pdf]

Figure 6B.

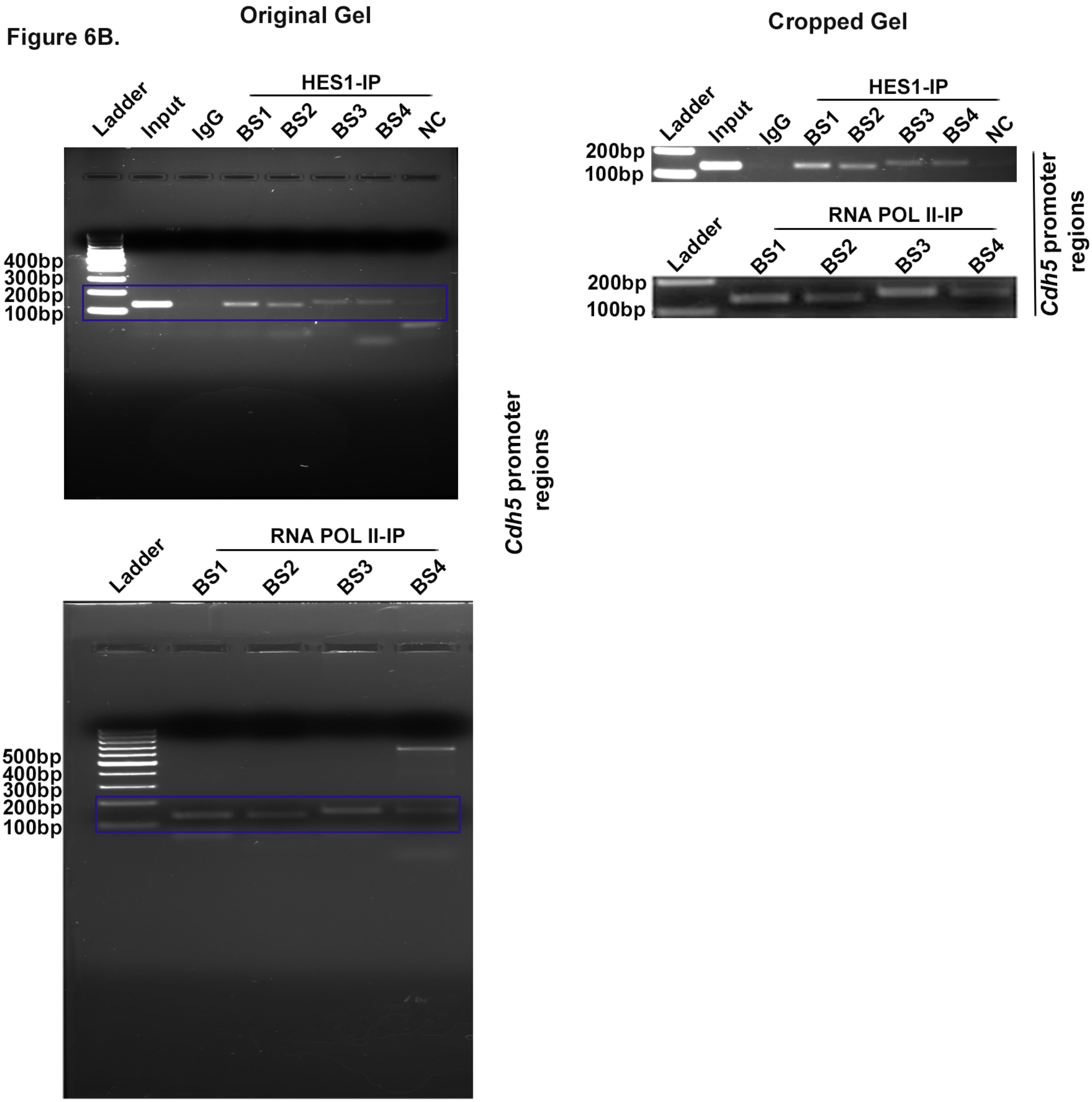
Original Gel
Cropped Gel
HES1-IP
Ladder
Input
IgG
BS1
BS2
BS3
BS4
NC
400bp
300bp
200bp
100bp
RNA POL II-IP
500bp
Cdh5 promoter regions

Figure 6D.

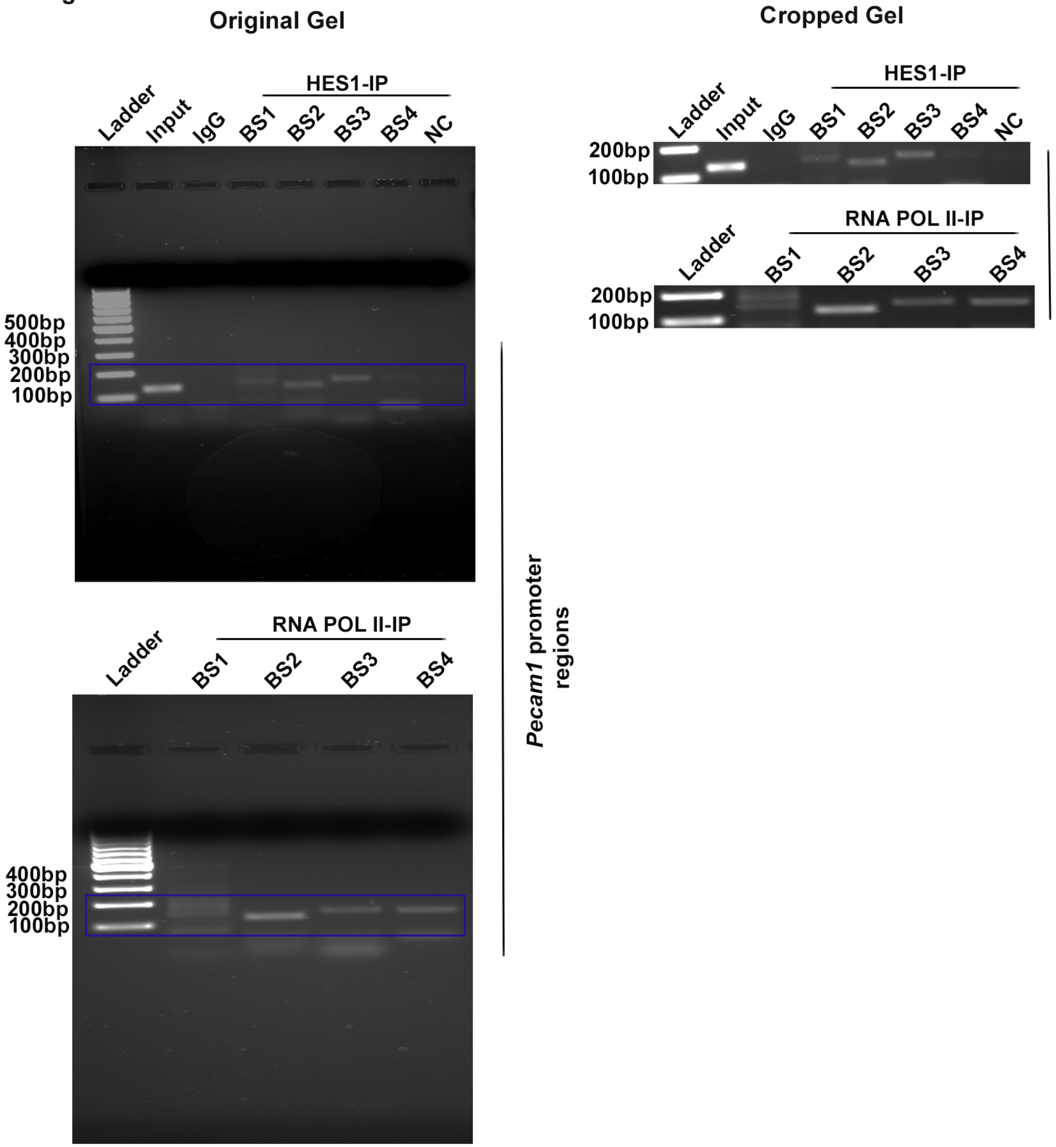

Original Gel
HES1-IP
Ladder
Input
IgG
BS1
BS2
BS3
BS4
NC
500bp
400bp
300bp
200bp
100bp
RNA POL II-IP
Ladder
BS1
BS2
BS3
BS4
400bp
300bp
200bp
100bp
Pecam1 promoter regions
Cropped Gel
HES1-IP
Ladder
Input
IgG
BS1
BS2
BS3
BS4
NC
200bp
100bp
RNA POL II-IP
Ladder
BS1
BS2
BS3
BS4
200bp
100bp

Figure 6F.

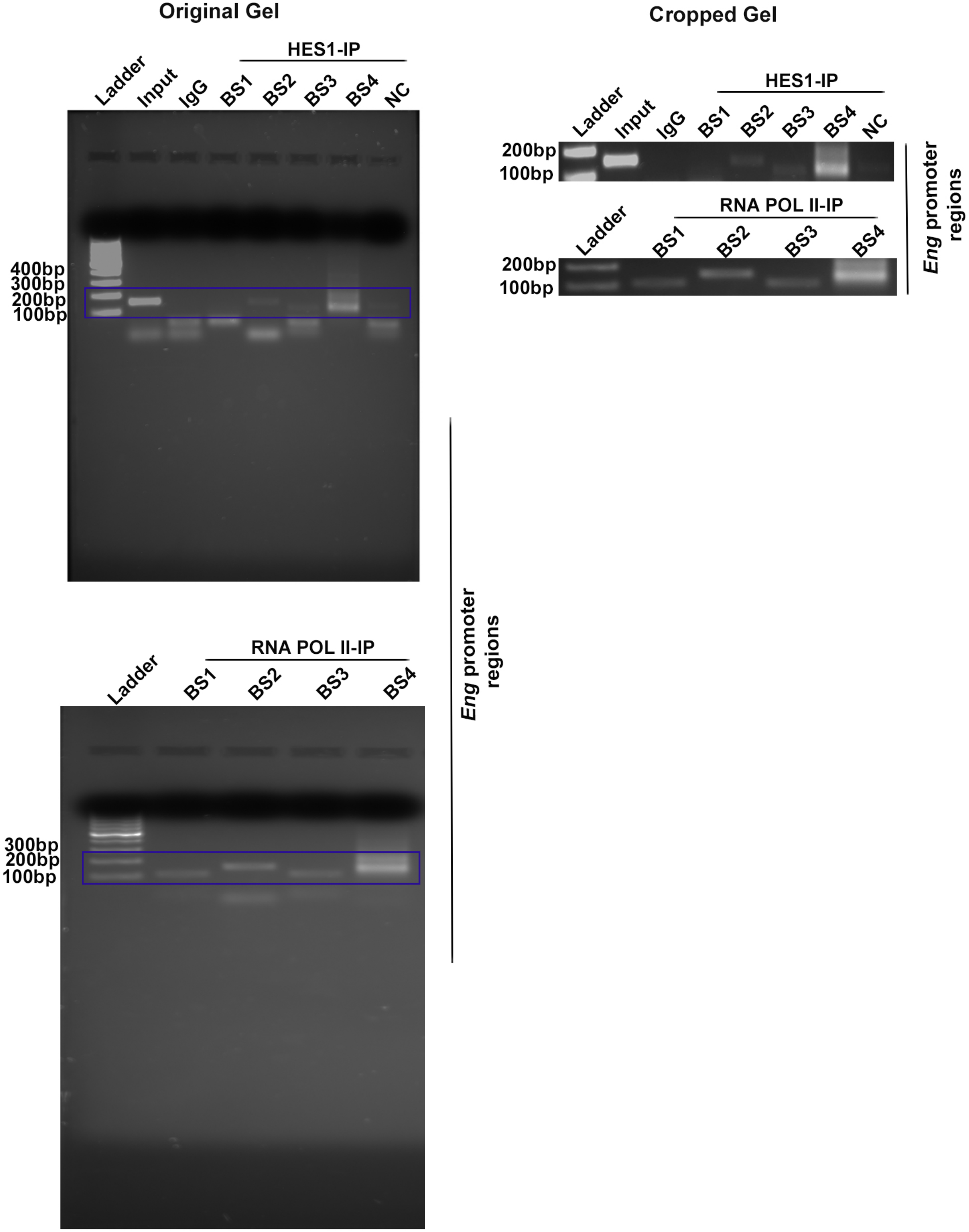
Original Gel
HES1-IP
Ladder
Input
IgG
BS1
BS2
BS3
BS4
NC
400bp
300bp
200bp
100bp
RNA POL II-IP
Ladder
BS1
BS2
BS3
BS4
300bp
200bp
100bp
Eng promoter regions
Cropped Gel
HES1-IP
Ladder
Input
IgG
BS1
BS2
BS3
BS4
NC
200bp
100bp
RNA POL II-IP
Ladder
BS1
BS2
BS3
BS4
200bp
100bp
Eng promoter regions
