# Supplementary material for: Trans-differentiation of trophoblast stem cells: implications in placental biology
Source: Life Sci Alliance. 2022 Dec 27;6(3):e202201583. doi: 10.26508/lsa.202201583 (PMC9797987; doi:10.26508/lsa.202201583)

**A.**

| | Apoptotic MS1 cells(%) | | | |
|---|---|---|---|---|
| | 0ng/ml | 6ng/ml | 12.5ng/ml | 25ng/ml |
| Replicate1 | 0.3 | 37.5 | 56.5 | 57.7 |
| Replicate2 | 0.4 | 38 | 58 | 59 |
| Replicate3 | 0.25 | 30 | 54 | 55 |

**D.**

| | Apoptotic MS1 cells(%) | |
|---|---|---|
| | 0h(control MS1) | 48h(cocultured MS1) |
| Replicate1 | 2.7 | 19 |
| Replicate2 | 4.6 | 14.3 |
| Replicate3 | 2 | 18 |

**E.**

Replicate1

Replicate2

Replicate3

| Apoptotic Diff cells(%) | | | |
|---|---|---|---|
| 0ng/ml | 6ng/ml | 12.5ng/ml | 25ng/ml |
| 0.5 | 0.9 | 0.5 | 0.3 |
| 0.4 | 0.8 | 0.9 | 0.85 |
| 1.1 | 1.4 | 1.3 | 1.9 |

| Apoptotic MS1 cells(%) | |
|---|---|
| 0h(control MS1) | 72h(cocultured MS1) |
| 0.1 | 30 |
| 1 | 40 |
| 2 | 18 |

## Original blots

**Figure 7F.**

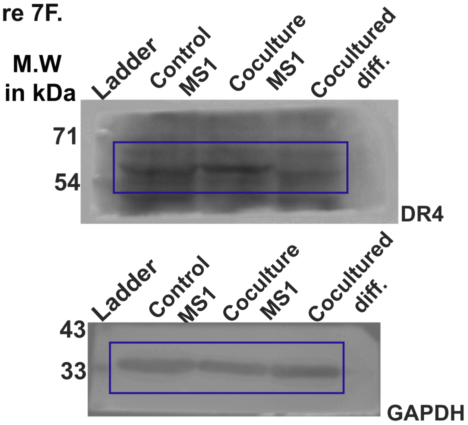


**Figure 7H.**

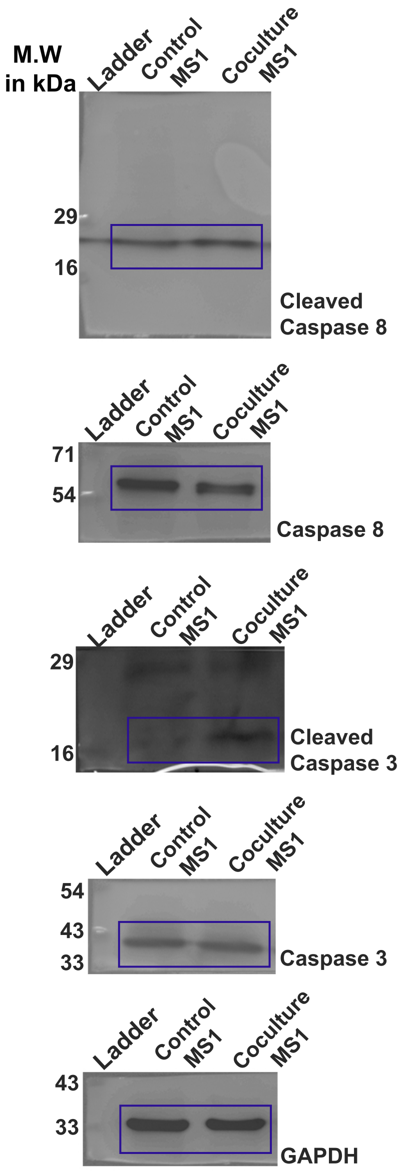


## Cropped blots

**Figure 7F.**

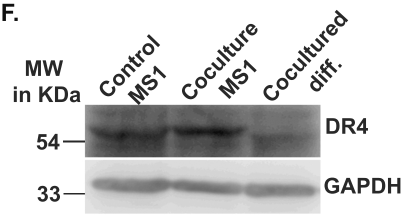


**Figure 7H.**

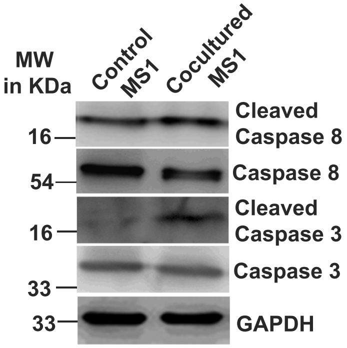

## Original blots

Figure 7J.

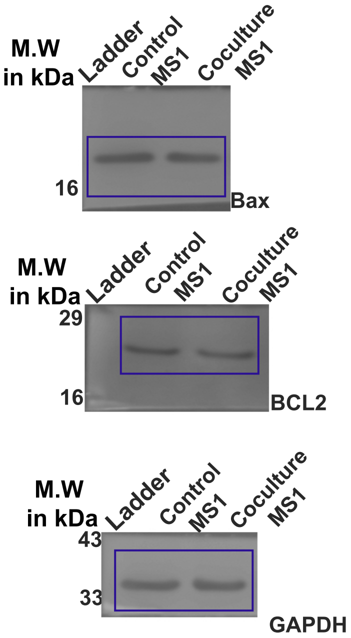


## Cropped blots

Figure 7J.

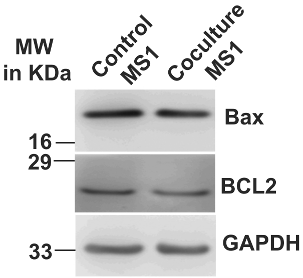

Supplement: Supplementary file 13 [file LSA-2022-01583_SdataF7.pdf]
