# Supplementary material for: Trans-differentiation of trophoblast stem cells: implications in placental biology
Source: Life Sci Alliance. 2022 Dec 27;6(3):e202201583. doi: 10.26508/lsa.202201583 (PMC9797987; doi:10.26508/lsa.202201583)
Supplement: Supplementary file 14 [file LSA-2022-01583_TableS2.docx]

**Table S2: Primer sequences used for real time PCR analysis**

|  | **Primer Name** | **Forward Primer Sequence**  **(5'-3')** | **Reverse Primer Sequence**  **(5'-3')** |
| --- | --- | --- | --- |
| 1 | *Cx3cl1* | CATTGTCCTGGAGACGACACA | TGTCCACCCGCTTCTCAAAC |
| 2 | *c-kit* | TTTACATAGACCCGACGCAACTT | TCATGGCAGCATCCGACTTA |
| 3 | *Kdr* | GTGAATGTCCCACCCCAGAT | CAGGCTTCTTCTAGCTGCCAGTA |
| 4 | *Plau* | ACTGCTTCATTCAACTCCCAAAG | TATGGTAGGCCAGGCTGTCTTC |
| 5 | *Mmp9* | CCAAAGACCTGAAAACCTCCAA | GCAGGGAGAGCTGCTTCTGA |
| 6 | *Cdh5* | TTCCAGCGACACTTCTACCACTT | TCGGAAGAATTGGCCTCTGT |
| 7 | *Pecam1* | CTCACGCTGGTGCTCTATGC | TTTCGAGGTGGTGCTGATGTC |
| 8 | *Eng* | CAGCAATGAGGTGATCATCAGTTT | GCCTAGTTCGATGGTGTTGGA |
| 9 | *Itgβ3* | GCGTGTCCCGTAATCGAGAT | TTCCATCCAGGGCAATATGG |
| 10 | *Col18a1* | AAGCTGACCTTCATTGACATGGA | TAGGAACCATTGATCCCAAAGC |
| 11 | *Tnsf10* | GGATCACTCGGAGAAGCAACTC | CTCGATGACCAGCTCTCCATTC |
| 12 | *Bcl2* | CGCCCTGTGGATGACTGAGT | GGCTGAGCAGGGTCTTCAGA |
| 13 | *Cradd* | CAGGAATTTCCCTGGGTAAGAGA | GCTGGTTAATCTGCTGGTCTGA |
| 14 | *Casp3* | GAGCTGGACTGTGGCATTGA | AACCACGACCCGTCCTTTG |
| 15 | *Rpl7* | AAGAAGCGGATTGCCTTGAC | TAACTTGAAGGGCCACAGGAA |
| 16 | *Hes1* | AAGAGGCGAAGGGCAAGAAT | AGGTGCTTCACAGTCATTTCCA |
