# Supplementary material for: Trans-differentiation of trophoblast stem cells: implications in placental biology
Source: Life Sci Alliance. 2022 Dec 27;6(3):e202201583. doi: 10.26508/lsa.202201583 (PMC9797987; doi:10.26508/lsa.202201583)
Supplement: Supplementary file 15 [file LSA-2022-01583_TableS3.docx]

**Table S3: Primer sequences used for ChIP PCR analysis.**

| **Target Name** | **Primer Name** | **Forward Primer Sequence**  **(5'-3')** | **Reverse Primer Sequence**  **(5'-3')** |
| --- | --- | --- | --- |
| *Cdh5* | BS1 | GAGGAGACTGAGGCATGGAT | TCAGATTCTTCCAGGTAGGGC |
|  | BS2 | TGCTATGAAGACCTCCACTGC | TGTACTCTAGCCCTGGTGGAT |
|  | BS3 | CCAGTACCTTCTCTGACTCCA | TGGCAGTGGAGGTCTTCATAG |
|  | BS4 | CAGGCAAGGCAGCAGTG | TGGAGACAGAATGGCTGGAG |
|  | NC | ACAGAACAGATTGTGGCAGAG | GCTCTGAGCGGAATCCATAG |
| *Pecam1* | BS1 | CACACACACAAACACACATACACA | GAACGCTACTGGTGGCTC |
|  | BS2 | CCTCACAAGGACTGCATATTGTCG | GGATACCTAGCAAGACCTGTTTGC |
|  | BS3 | AGGCAGAAACCATTAAGGCAG | GGTCACATTGTGAGACTATGTCC |
|  | BS4 | GTGCCTCTACCACCAGAACC | GCAAGAGGGTCAGAGGTTAAAGG |
|  | NC | GACTTATGTGGTGATCAATGGTGG | TACAGAGCAAGTTGTAGGACAGC |
| *Eng* | BS1 | TACAGGGACTTCAACCAGACCTT | AACAGACCTCCCCCATGC |
|  | BS2 | GGCTAAGGCATGGACTTAGAAAG | GGCGAGATGACAGGCAG |
|  | BS3 | GCCTCCTGGGACATTTAGTGA | CCTAAGGGAATGAAGGGTCTGG |
|  | BS4 | ATGTGGTTGCTGGGATTTGAAC | ACACACACACACACACACTAATG |
|  | NC | GCCTTCTCAAGAGAGATGACTCC | CAGCAAGCAAAGAACCAAGCATA |
